# Evaluating the impact of a peer-education digital literacy course on older adults' digital skills and wellbeing: a mixed-methods study protocol

**DOI:** 10.3389/fsoc.2024.1432607

**Published:** 2024-07-09

**Authors:** Dario Pizzul, Emanuela Sala, Alessandro Caliandro, Daniele Zaccaria, Simone Carlo

**Affiliations:** ^1^Department of Political and Social Sciences, University of Pavia, Pavia, Italy; ^2^Department of Sociology and Social Research, University of Milano-Bicocca, Milan, Italy; ^3^Department of Business Economics, Health and Social Care, University of Applied Sciences and Arts of Southern Switzerland (SUPSI), Manno, Switzerland; ^4^Faculty of Political and Social Sciences, Catholic University of Milan, Milan, Italy

**Keywords:** digital education, peer education, digital literacy course, older adults, aging, digital skills, wellbeing

## Abstract

The digital transition poses relevant challenges and opportunities for older adults in aging European societies. To unleash the potential of the digital transition in old age and avoid the risk of exclusion, digital education for older adults seems to be a valuable solution. One of the most suitable approaches to digital education for older adults appears to be the peer-to-peer approach. However, not much literature is available on this topic. Within the ACTIVE-IT project, we aimed to design, implement, and evaluate a digital peer education course for older adults, focusing specifically on the use of smartphones and daily utility apps, such as mailing, e-Gov, and e-commerce. The purpose of this contribution is to document the protocol adopted to evaluate the course. The course involved 32 participants aged 65 or older, who, between March 2024 and June 2024, divided into three groups, attended a 10-lesson weekly course taught by a peer. We aim to measure the effect of the course on participants' digital skills and their perceived wellbeing. To do so, we will adopt a mixed methods approach, employing: digital methods by collecting and analyzing data on participants' smartphone use (i.e., log data on smartphone use before/during/after the intervention); a quasi-experiment, collecting information on course participants' wellbeing before/after the course attendance using a questionnaire survey; ethnographic observation conducted during the course, observing interactions between subjects during the course. The study has been approved by the Ethic Committee of the University of Milano Bicocca (prot.nr. 167541/2024).

## 1 Introduction

Europe stands out globally for having one of the highest proportions of older adults in the population. Within the European Union (EU), Italy leads with the highest percentage of individuals aged 65 and above. As of 2022, Eurostat reported that 23.8% of Italy's population fell within this age group (Eurostat, 2023a). Furthermore, projections indicate a continued increase in the older adults population over the coming decades (Eurostat, 2023b). The aging of contemporary societies presents new challenges to the economic sustainability of welfare systems (European Commission, 2020). At the same time, safeguarding older adults' wellbeing and social inclusion and valorizing their contribution represents an opportunity to reinforce the social and economic sustainability and equity of society as a whole (Rossi and Scabini, 2016). Examining population aging in the context of the ongoing digital revolution reveals additional challenges and opportunities. On one hand, with only 28.5% of individuals aged 65–74 in the EU possessing basic or higher digital skills (Eurostat, 2023c), such as the ability to use devices like smartphones and computers and access the Internet to manage information. This creates a significant risk of exclusion from an increasingly digital society among older demographics, a risk that is less present in other age groups due to their higher digital skills (Eurostat, 2024). Notably, Italy's figures are even lower, with only 19.3% of 65 to 74-year-olds and just 4.6% of those aged 75 and above having basic or higher digital skills (Eurostat, 2023c). On the other hand, it has been argued that digital technologies may contribute to reducing older adults' social exclusion, increasing chances to connect with families, friends, or service providers (Baker et al., 2018; Sala and Gaia, 2019). This connectivity can also have a positive impact on wellbeing, a multifaceted dimension among older adults (Saadeh et al., 2020), which is strongly related to happiness and life satisfaction (ESS, 2024).

Digital education is a known strategy to tackle these challenges and harness the potential of the digital transformation in older age. There are numerous examples of digital education programs targeted at older adults, often employing the reverse mentoring model. This approach involves younger instructors, such as college students, teaching older individuals specific digital skills (Breck et al., 2018; Leek and Rojek, 2023). However, growing skepticism surrounds its efficacy due to concerns that younger mentors may lack patience and fail to understand the unique needs of older learners, who may also be hesitant to learn from younger instructors (Xie, 2007; Naumanen and Tukiainen, 2010; Carlo and Bonifacio, 2020). Peer-to-peer digital education, where digitally savvy older adults teach their peers, may overcome some of the limitations of the reverse mentoring approach and could be an effective method to teach older adults the skills needed in an increasingly digitized world.

This paper aims to document and detail the protocol used to measure the impact of a digital peer-education course on both digital skills and perceived wellbeing in 32 older individuals. As a study protocol, this contribution does not aim to report results; these will be presented once the study has been conducted. To our knowledge, this is the first study of its kind addressing an underexplored yet significant issue. Even though our study is not a clinical trial, we referred to the SPIRIT (Standard Protocol Items: Recommendations for Interventional Trials) guidelines as much as possible while writing this protocol (Chan et al., 2013). This ensured that we followed a defined reporting grid to present our protocol.

## 2 Peer education among older adults

Peer education among older adults is a well-established approach in the healthcare domain (Buonocore and Sussman-Skalka, 2002; Ghasemi et al., 2018). There are also valid reflections on the best approaches to structuring peer education courses (Brady et al., 2003; Choi, 2009). However, concerning older adults' peer teaching of digital skills, the literature is very limited (Rasi et al., 2021). In reviewing different approaches to promoting media literacy among older adults, different authors mention the existence of peer education without delving into it (Rasi et al., 2021; Ahmad et al., 2022), mainly due to the shortage of published research. In fact, there are not many contributions describing digital peer education courses' features, evaluating their effectiveness, and measuring their implications for older adults' wellbeing.

The systematic exploration of 18 academic databases (EBSCO AgeLine/Communication Abstracts/Education Research Complete, ProQuest APA PsycArticles/APA PsycInfo/Periodicals Index Online/Publicly Available Content Database/Education Database/ERIC/International Bibliography of the Social Sciences/Social Science Database/Applied Social Sciences Index & Abstracts/Social Service Abstracts/Sociological Abstracts/Sociology Database, Pub Med, Scopus, Web of Science), conducted using keywords related to aging, peer education, and ICTs, resulted in 393 contributions written in English. However, most of them did not align with our interests, primarily due to a lack of coverage on the old age population, their digital education, or the absence of references to peer-to-peer approaches. Only four papers more or less extensively discussed peer-to-peer digital literacy courses' characteristics and methods used to evaluate their effectiveness and their impact on wellbeing (Russell, 2011; Woodward et al., 2013; Burmeister et al., 2016; Pihlainen et al., 2021). The format of the courses varied significantly: Woodward et al. (2013) discuss a 19 people course articulated in 20 meetings; Burmeister et al. (2016) and colleagues worked with six older adults following 16 classes, while Russell (2011) covers a course lasting a full year with 16 participants. Seemingly, the different authors employed different approaches to evaluate the peer course effects on wellbeing, using qualitative approaches (Russell, 2011; Burmeister et al., 2016) or open-ended questions in a survey (Pihlainen et al., 2021). Leveraging a quantitative approach, Woodward et al. (2013) employed four scales for evaluating participants characteristics: the Multidimensional Scale of Perceived Social Support to measure perceived social support; a six-item scale to measure loneliness; a 16-item scale to measure quality of life; and the Geriatric Depression Scale for assessing depressive symptoms. Woodward et al. (2013) also employed the Computer Self-efficacy Murphy Scale to measure how participants' perception of their skills varied before, during, and after the course.

These four papers (Russell, 2011; Woodward et al., 2013; Burmeister et al., 2016; Pihlainen et al., 2021), still having the advantage of covering the largely unexplored issue of digital peer education among older adults, leave many dimensions of the matter uncovered. For this reason, we aim to design, implement, and evaluate, based on the following protocol, a peer-to-peer digital literacy course for older adults that could contribute to deepening the knowledge on this important issue. The course primarily focuses on the use of smartphones and daily utility apps, such as mailing and communication services, e-Gov, e-commerce, mobile payments, and mobility. It has been co-designed and will be implemented in collaboration with AUSER Monza-Brianza (AUSER MB), a non-profit organization for active aging based in Monza, a city in the northern Italian region of Lombardy. The course, comprising 10 weekly face-to-face sessions each lasting one and a half hours, will be delivered three times, involving overall 32 participants [two groups of nine older adults, attending classes in the city of Monza (IT) and one of 12 attending classes in the city of Nova Milanese (IT)].

## 3 Methods and analysis

### 3.1 Overview of the study design

Several scholars have reflected on the evaluation process of educational interventions among older adults (Xie, 2011; Esteller-Curto et al., 2015; García-Camacha et al., 2019). Some have specifically focused on the effects on the quality of life after the intervention (Shapira et al., 2007; Esteller-Curto and Escuder-Mollon, 2012; Woodward et al., 2013), while others have concentrated on the skills and competencies developed through the courses (Xie, 2011; Esteller-Curto and Escuder-Mollon, 2012). Building on these contributions and aiming to incorporate both perspectives, we employ a mixed-method approach based on the following three research methods:

- the collection and analysis of digital data on participant smartphone use (i.e., log data from the application Stay Free—Screen Time, monitoring course participants' smartphone use before/during/after the intervention);
- the realization of a quasi-experiment, collecting information on course participants' wellbeing before/after the course attendance using a questionnaire survey;
- the realization of an ethnographic observation conducted during the course, observing interactions between subjects during the course.

The details of the three research methods employed to perform the evaluation of the peer-to-peer digital literacy course are described in Section 3.5.

### 3.2 Study participants and sample size calculation

Study participants are 32 (20 women and 12 men) individuals who will attend a digital education course (the same course is delivered to three different groups of 9, 9, and 14 individuals). The inclusion criteria to participate in the digital peer education courses are 2-fold: being 65 years or older and owning a smartphone. In defining the sample size, we were driven both by our literature review, previous AUSER MB experience in delivering similar courses, and practical reasons. Specifically, the literature review has documented that the peer-to-peer digital education courses are normally delivered to small-size classes, involving 6, 16, and 19 participants (see, respectively, Russell, 2011; Woodward et al., 2013; Burmeister et al., 2016). We finalized the number of participants attending each course (approximately ten course participants) by consulting previous AUSER teachers who have delivered similar courses in the past and evaluating the characteristics of the available teaching rooms (e.g., number of computers: nine in one room). To ensure variability in the research findings, we decided to organize three courses.

### 3.3 Recruitment and informed consent process

AUSER MB oversaw the recruitment of the course participants among its affiliates or people who in general participate in their activities. The recruitment started in January 2024. We provided AUSER MB with information materials about the study, i.e., a two-pages leaflet with a presentation of the study, to be used during the recruitment stage.

Study participants underwent an informed consent process. The overall aim of this process was to inform course participants about the study's purposes and data collection, usage, and storage process. In designing the consent process, we draw on our previous experiences when delivering similar courses (Caliandro et al., 2021), the provided guidelines by the Ethical Committee of the University of Milan—Bicocca, as well as other studies employing a similar research approach (Kreuter et al., 2020; Stier et al., 2020). One month before the start of the digital education courses, in February 2024, we organized a kickoff meeting with potential course participants, divided in three groups. In the kickoff we presented the study and introduced the research team^1^, provided each course participant with the information document, explain the informed consent process, collected the signed informed consent after having answered all the questions. Upon having collected the signed consent forms, we asked course participants to install the Stay Free—Screen Time app on their smartphones, providing assistance in case of need.

### 3.4 Intervention

The intervention involves delivering a peer-to-peer digital education course on the use of smartphones and daily utility apps. This course has been co-constructed between September 2023 and March 2024 in collaboration with AUSER MB. This implies that the characteristics of the course participants and peer educators, the teaching method, as well as the course contents, were discussed and agreed upon by AUSER MB and the research team. The digital literacy course comprises 10 weekly classes, each lasting one and a half hours. The same course is delivered three times, twice at an AUSER MB venue in Monza and once at an AUSER MB venue in the nearby city of Nova Milanese. The teaching is conducted by two male peer educators—specifically, digitally savvy individuals from the same age class, namely 65 years old or older. One of the peer educators has previous experience in teaching peers, as he teaches another basic computer course that AUSER MB usually offers to its affiliates. This peer educator oversees the two courses taking place in Monza, while the one in Nova Milanese is conducted by another peer educator with some teaching experience in digital topics, gained during his occupation before retirement. It is important to state that neither of them are professional digital educators and they carry out this activity as volunteers. As previously mentioned, a member of the research group is present during classes at all three venues.

The course aims to enhance participants' digital skills concerning the use of smartphones, specifically focusing on daily utility apps such as email apps, eGov apps, mobility apps, etc. The course is structured into the following ten 90-min lessons:

1. From Computer to Smartphone: Emails—part 1
2. From Computer to Smartphone: Emails—part 2
3. Internet and Online Accounts—part 1
4. Internet and Online Accounts—part 2
5. Online payments and shopping—part 1
6. Online payments and shopping—part 2
7. Frequently used apps: download and use
8. Public Administration: e-Gov
9. Public Administration: healthcare
10. Frequently used apps

The intervention started in March 2024 and lasted until June 2024 for 10 non-consecutive weeks (due to Easter festivities).

### 3.5 Assessments

This study aims to assess the peer-to-peer digital literacy course, both by evaluating its impact on participants' digital skills (Esteller-Curto and Escuder-Mollon, 2012) and the effect on their wellbeing (García-Camacha et al., 2019). In short, we will evaluate the effect of course attendance on participants' actual smartphone using patterns (i.e., whether participants will use certain apps more extensively after the intervention, whether they will use new apps that they did not use before, etc.) which should be informative about their digital skills, and the impact of the course on perceived wellbeing. We are also interested in investigating what kind of social interactions develop during the course (between study participants and between study participants and the educator) and how they impact the learning processes. As previously mentioned, to perform the assessment of the digital literacy course, we developed a procedure based on a mixed-method approach. The overview of the assessment is available in Table 1.

**Table 1:** Overview of assessments.

**Assessment aim**	**Methods & measures**	**Timing**			
		**February 2024 kick off**	**March 2024 first lesson**	**June 2024 last lesson**	**June 2024 end**
To evaluate the impact of the course attendance on smartphone usage patterns	• Digital methods • Primary data collection/analysis of log data from Stay Free - Screen Time app: ° Names of the apps used ° Exact time (day, hour, minutes) the apps started/stopped to be used ° Duration of use	Measurement starts	Measurement continues	Measurement continues	Measurement ends
To assess the impact of the course attendance on course participants wellbeing	• Questionnaire survey • Primary data collection/analysis of an online short questionnaire administered twice: ° Current use of and competence on digital device use (MDPQ-16 scale) ° Attitudes toward technology (Anderberg et al., 2019) ° Life satisfaction, happiness and Wellbeing [Technostress scale (Nimrod, 2018); Happiness and Life Satisfaction measures (ESS, 2024)] ° Demographic and socio-economic characteristics ° Health conditions		First self-compilation	Second self-compilation	
To identify strength and weaknesses of the peer-to-peer digital literacy course evaluation	• Ethnographic approach • Primary data collection/analysis of observational field notes: no specific standardized and quantitative measures		Start of observation	End of observation	

#### 3.5.1 Evaluating the impact of the peer-to-peer digital education course on smartphone using patterns

To evaluate changes in smartphone usage patterns, we employ digital methods to collect data from participants' phone activities. Specifically, we will assess the actual use of the smartphone (Boase and Ling, 2013; Rosales and Fernández-Ardèvol, 2016; Kreuter et al., 2020; Stier et al., 2020), collecting smartphone usage data for about 4 months: (1 month) before the start of the course, during the course attendance, and (1 month) after the course has ended. Our aim is to empirically compare course participant usage patterns before and after the intervention, monitoring the advancements achieved in the meanwhile. In practice, after having collected the written consent form, during the kickoff meeting participants were invited to install the app The Stay Free—Screen Time app onto their smartphones. Additional details on The Stay Free—Screen Time App are provided in Section 3.6.

#### 3.5.2 Evaluating the impact of the peer-to-peer digital education course on participants' wellbeing

To evaluate the impact of the peer-to-peer digital education course on course participants' wellbeing, we will adopt a quasi-experiment approach, inviting course participants to fill in a short online self-completed questionnaire during the first and last class of the course. Details on the measures included in the questionnaire are presented in Section 3.6.

#### 3.5.3 Investigating interactions occurring during the course

To highlight strengths and weaknesses of the designed course, ultimately improving the protocol on the peer education course, we will adopt an ethnographic approach observing interactions occurring during the course lessons (of the three courses). In practice, one of the researchers will attend all classes, taking notes on participants' interactions. The observation will focus on three dimensions:

- The interactions between participants;
- The interactions between participants and the peer educators;
- The interactions between participants and technological devices.

It should just be pointed out that the employed approach largely refers to the “focused ethnography” one (Knoblauch, 2005; Strada et al., 2013). This method relies on short-term field visits as opposed to long-term ones of more classical approaches. Within such an approach, the researcher covers the role of the field observer rather than the participant. Moreover, the main focus is on the communicative activities of participants and not the social field around them (Knoblauch, 2005). Still, the used approach does not fully qualify as “focused ethnography” since no video recording of the teaching sessions will be performed. The research team decided not to perform video recordings of participants since they could have perceived them as too invasive, especially because of the other measurements performed on their smartphones.

### 3.6 Measures

As presented in Table 1, we will use several different measures, suitable to address the three specific research aims.

#### 3.6.1 Evaluating the impact of the peer-to-peer digital education course on smartphone using patterns

Monitoring participants' smartphone usage through the Stay Free—Screen Time app will collect the following three objective measures (of smartphone usage) for each participant, who will be identified with an alphanumeric ID:

- names of the categories of the apps that participants used during the monitoring period (4 months);
- The exact time (day, hour, minutes) the categories started/stopped to be used;
- The duration (in seconds) of use of each app.

As previously mentioned, such information will provide objective evidence on smartphones' use by participants, as shown in several studies (Boase and Ling, 2013; Rosales and Fernández-Ardèvol, 2016; Kreuter et al., 2020; Stier et al., 2020). It is important to clarify that by no chance it will be possible to access the content of the activities performed on the apps' categories. For instance, the Stay Free—Screen Time app registers that a participant used a navigation app on April 22nd, 2024, at 4 p.m. for 25 min, but it does not register the address that s/he searched in the app, nor the location, or any additional features. The Stay Free—Screen Time app was developed by Sensor Tower^2^. Sensor Tower is an U.S.-based company specialized in market intelligence and performance metrics in the mobile app and digital advertising ecosystem. It provides many services to several customers in the brands, gaming, finance, and advertising industries. The Stay Free—Screen Time app is one among the apps that the company has developed. The app usually is used by users as a self-monitoring tool for measuring own activities on devices and improving self-management. As happened in other contributions (Rosales and Fernández-Ardèvol, 2016), we have repurposed the affordances of the app for academic research purposes. The data will be supplied to us by the company at three different moments: midway through the course, at the conclusion of the course, and 1 month after its completion. Therefore, it will be possible to assess if any changes in smartphone usage behavior occurred after the intervention.

#### 3.6.2 Evaluating the impact of the peer-to-peer digital education course on participants' wellbeing

By means of a questionnaire survey, we will collect the following five types of measures:

- Current use of and competence on digital device use [MDPQ-16 scale (Roque and Boot, 2018)]
- Attitudes toward technology (Anderberg et al., 2019)
- Life satisfaction, happiness and Wellbeing [Technostress scale (Nimrod, 2018); Happiness and Life Satisfaction measure (ESS, 2024)]
- Demographic and socio-economic characteristics
- Health conditions

MDPQ-16 is a validated scale, specifically developed to measure mobile device proficiency in older adults. It is a short 16-questions version of the Mobile Device Proficiency Questionnaire (MDPQ) which presents to respondents several digital related tasks, from turning on and off the device to deleting games and application, and asks them how confident they are in performing such tasks (Roque and Boot, 2018). Members of our research team used this measure in another study (Rolandi et al., 2022).

To measure attitudes toward technology, we used a 6-item scale from the work “A Novel Instrument for Measuring Older People's Attitudes Toward Technology (TechPH): Development and Validation” by Anderberg et al. (2019). Participants are asked to express their level of agreement to propositions such as: “using technology makes life easier for me” or “I like to acquire the latest models or updates”.

Life satisfaction and happiness level are standard measures and are collected using a ten-point Likert scale, as done in the European Social Survey. Specifically, one question asks: “taking all things together, how happy would you say you are?”, while the other “all things considered, how satisfied are you with your life as a whole nowadays?” (ESS, 2024).

To measure wellbeing, we referred to the work of Nimrod (2018). She specifically focuses on older adults, proposing a 14-item scale to measure technostress which is a threat to wellbeing in later life. The scale asks to agree or disagree with a number of statements related to activities related to digital devices, such as “This technology makes me do things slower” or “I often find the technology too complex to use”.

Demographic and socio-economic characteristics are standard measures in socio-economic questionnaire surveys (e.g., Survey for Health, Aging and Retirement in Europe) and include questions about gender, level of education, previous job position, marital status, leaving conditions whether respondents live alone or not. The exact questionnaire wording is reported in the questionnaire.

Health conditions are measured with a standard self-assessment of participants' general health conditions.

#### 3.6.3 Investigating interactions occurring during the course

As ethnographic studies do not usually entail the collection of “standardized” and “quantitative” measures, we will focus on interactions between participants, participants and the peer educators, and participants and technological devices. For example, we will specifically observe whether participants discuss certain topics presented during classes, seek mutual support, or ask questions to the peer educator and on what kind of topics. Additionally, we will observe whether they experience any difficulty in interacting with mobile devices, which are the most complicated tasks to perform. Furthermore, we will consider how they engage with the teaching process, whether they take notes, just listen, or ask several questions, etc. Furthermore, rather than solely previous methodologies to measure the effect of the course on digital skills and perceived wellbeing, we use participant observation to contextualize quantitative data—specifically, tracking and survey data. As well-acknowledged in the literature, tracking data do not speak for themselves; they can sometimes be very ambiguous (Ferreira et al., 2014). To properly interpret tracking data, researchers need to rely on qualitative methods (Aipperspach et al., 2006; Ørmen and Thorhauge, 2015; Caliandro et al., 2021). For example, an increased frequency in the use of a maps app registered by the Stay Free software does not necessarily mean that participants improved their capacity in using the app; it could simply mean that they logged into the app more often under the instructor's exhortation. Therefore, participant observation during the courses can definitely help to shed light on such ambiguous cases, as the researcher will have the opportunity to both observe and talk to participants during their learning process. A similar scenario applies to survey data. Survey data are not ambiguous *per se*, but it is always useful to triangulate survey data with qualitative data to achieve a more nuanced and in-depth understanding of a given phenomenon, especially when working with small samples, which make comparisons and double checks much easier (Bryman, 2016). In our case, it will be particularly interesting to compare the quantitative measurement of participants' wellbeing with their subjective ideas of what wellbeing in relation to the usage of digital technologies is.

### 3.7 Data analysis plan

Since the study employs three different methods, the data analysis plan is 3-fold.

On the one hand, we will analyze log-data from the Stay Free—Screen Time app measurement to provide a detailed picture of participants' smartphone usage patterns and detect changes over time, possibly due to the attendance to the peer-to-peer digital education course. We will mainly use descriptive statistical analysis (e.g., categorization of the most used apps, average time of use for the most recurrent apps, and the time of day when participants used their phone the most). Data will be analyzed using Python.

Then, we will analyze quantitative data from the questionnaire survey administered to 32 course participants using both descriptive and multivariate analyses. Our primary approach will be to use regression discontinuity design (RDD) to establish the causal effect of the intervention. However, given the exploratory nature of our study and the constraints on sample size discussed with our partner AUSER MB, the study may be underpowered for such advanced econometric models. To address this, we will use robust standard errors to account for heteroscedasticity, apply bootstrapping methods to enhance the reliability of our estimates, and conduct sensitivity analyses to assess the robustness of our findings. If RDD proves to be infeasible due to the small sample size, we will consider alternative analyses such as difference-in-differences (DiD) or mixed-effects models, which are more appropriate for smaller samples. Furthermore, to manage potential missing data from participant dropout, we will use multiple imputation techniques and perform intention-to-treat (ITT) analysis to maintain the integrity o our findings.

Lastly, field notes from the ethnographic observation (of the 10 classes of each of the three courses) will be analyzed using the software NVivo, performing qualitative analysis. We will transform the field notes, gathered during all the 30 lessons, into concepts (Carlo and Bonifacio, 2020). We then further group concepts into themes, following a thematic analysis approach, in order to interpret the notes on the observed interaction.

## 4 Ethics and dissemination

We have developed a dissemination plan aimed at engaging both team members and the project stakeholders (i.e., AUSER MB and peer educators) in all stages of the research project, from its design to the dissemination of the research findings. Indeed, the peer-to-peer digital literacy course was co-designed together with AUSER MB. The research groups and AUSER MB extensively discussed the study and the related measures to be collected in several meetings. Course participants received full information about the study and the research approach adopted to evaluate its effectiveness. They received an oral and written presentation of all the features of the study and had the chance to ask questions to the research team.

Once the course and the empirical measurements will be over, we plan to share the preliminary research findings with the participants, peer educators, and AUSER MB via in-person meetings. Subsequently, academic conference presentations and scientific publications on the findings will follow. Lastly, this protocol received approval by the Ethical Committee of University of Milan—Bicocca (prot.nr. 167541/2024).

## 5 Conclusions

Peer-to-peer digital education among older adults could be a valuable solution to the increasing digitization of society and the challenges that it poses to social inclusion. However, there is very little knowledge about the most appropriate features of digital peer education courses, their effectiveness on digital skills, and their implications for the wellbeing of older adults. Our protocol aims to establish a research strategy based on a mixed-methods approach to evaluate the impact of a peer education digital literacy course among older adults. Relying on digital methods through smartphone activity monitoring, a quasi-experimental design via a questionnaire survey, and an ethnographic observation, we have outlined a novel protocol that may enhance our knowledge on this topic. Due to its explorational nature, our project has the main limitation of being highly context-specific and, therefore, not generalizable. Furthermore, the limited number of participants makes the potential impact of dropout even more significant. The population of interest could also lead to increased chances of dropouts due, for example, to health problems, as seen in other studies (Burmeister et al., 2016). Clearly, these are events that cannot be controlled by the research group, but it is important to acknowledge that these potential limitations exist. Future studies may consider applying our evaluation protocol to larger and more statistically relevant samples of older adults attending a peer education digital literacy course.

## Ethics statement

The studies involving humans were approved by Ethic Committee of the University of Milano Bicocca (prot.nr. 167541/2024). The studies were conducted in accordance with the local legislation and institutional requirements. The participants provided their written informed consent to participate in this study.

## Author contributions

DP: Writing – original draft, Writing – review & editing. ES: Writing – original draft, Writing – review & editing. AC: Writing – original draft, Writing – review & editing. DZ: Conceptualization, Writing – review & editing. SC: Conceptualization, Writing – review & editing.

## Footnotes

^1^DP and AC attended the kickoff meeting and introduced the other members of the group (ES, DZ, SC), who originally contributed to conceptualizing the research. DP was then the researcher attending all classes.

^2^ https://sensortower.com/
